# Chronic Early Life Stress Alters the Microbial and Transcriptional Profile of the Zebrafish Gut

**DOI:** 10.21203/rs.3.rs-7491371/v1

**Published:** 2025-10-31

**Authors:** Erik Norloff, Katherine Coker, Samir Tusneem, Cameron T. Dixon, Karen Zhu, Christina L. Graves

**Affiliations:** University of North Carolina at Chapel Hill; University of North Carolina at Chapel Hill; University of North Carolina at Chapel Hill; University of North Carolina at Chapel Hill; University of North Carolina at Chapel Hill; University of North Carolina at Chapel Hill

**Keywords:** Early life stress, chronic stress, zebrafish, microbiota, gut, mucosal immunity

## Abstract

Chronic early life stress (ELS) is appreciated to potently shape a myriad of biological outcomes later in life and has been associated with fertility deficits and the onset of gastrointestinal dysfunction in humans. Further, recent longitudinal cohort studies demonstrate that multigenerational adversity impacts the gut microbiome composition in early childhood, highlighting the gut-brain axis as an important target of ELS. Building on our recently published work demonstrating that ELS alters the neuroimmune profile of the developing zebrafish gut, our goal here was to establish a model of multigenerational ELS in zebrafish and determine cumulative stress impacts on fertility, gut microbial composition and the transcriptional landscape of the developing gut. Wild-type zebrafish were exposed to chronic ELS beginning at 5 dpf until 30 dpf according to our recently published stress paradigm for a total of four successive generations. We compared stressed and unstressed groups from either stressed or unstressed lineages and found that chronic ELS was associated with reduced egg viability and profound changes to the gut microbiome. RNA-sequencing revealed ELS-associated differential expression of more than 800 genes in founder generations. Altogether our data demonstrate that zebrafish are a powerful model for exploring neuroimmune interactions at mucosal surfaces across generations.

## Introduction

Early life stress (ELS) is appreciated to illicit profound changes to human health and behavior, with persistent effects throughout development and into adulthood. We recently developed a novel model of chronic ELS in zebrafish and demonstrated that ELS results in dramatic changes to the developing gut including differential expression of neuroimmune-related genes^1^. In addition, mounting experimental and observational evidence indicate that ELS remodels gut microbial composition, although the specific changes appear to be context-dependent and the underlying mechanisms mediating these links remain incompletely understood^2,3^. In addition to impacts to the neuroimmune and microbial-gut-brain axis^4^, emerging evidence also indicates that ELS impairs reproductive function and success, potentially through a variety of mechanisms affecting both male and female sexes^5–8^. Here, recent studies have demonstrated that the gut microbiota can influence gonadal and reproductive function through diverse pathways highlighting the existence of a gut microbiota-gonadal axis^9,10^. Despite these compelling associations, underlying tissue-specific mechanisms remain underexplored. In this study, we interrogated the effects of chronic ELS on egg viability and survival, as well as the impact on gut microbial composition through a generational lens. We further investigated ELS-associated effects on the gut transcriptional landscape to explore mechanisms by which ELS impacts the gut-microbiota-gonadal axis.

## Methods

### Animal Husbandry

**Figure 1:**
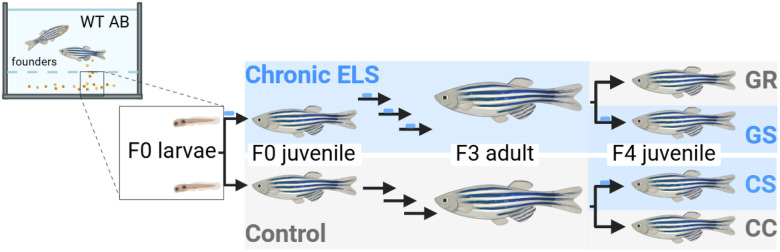
Overview of the Experimental Design. Offspring from WT AB founder fish were subjected to a chronic ELS paradigm as previously published (Graves *et al*. 2023). Subsequent generations were also subjected to this ELS paradigm for a total of 4 generations. In the fourth generation, fish were subjected to ELS with (GS) or without (CS) prior generational exposure and compared to control fish with (GR) or without (CC) prior generational exposure. For all ELS experiments, ELS was administered ~5–30dpf).

All experimental procedures were reviewed and carried out with the approval of the UNC Chapel Hill IACUC (Protocol #20–241, #23–178) and following ARRIVE guidelines^11^. All experiments were performed in accordance with relevant guidelines and regulations. Wild-type (AB) zebrafish were reared and maintained in the AAALAC-accredited UNC Zebrafish Aquaculture Core Facility under a 14-hr light/10-hr dark cycle at 28°C. For stress induction, WT AB zebrafish were subjected to a chronic early life stress (ELS) paradigm as previously published^1^. Experiments are designed as a two group (ELS [CS] vs. control [CC]) or four group comparisons. For four group comparisons fish with and without prior generational ELS exposure were included, referred to as “generational recovery” (GR) and “generational stress” (GS), respectively, as shown in Fig. 1. Experimental animals were determined by tank and randomly assigned. The total number of animals used for each experiment is included in the corresponding methods and figure legends; no animals were excluded from analysis. Anesthesia and euthanasia was performed using tricaine immersion (500 mg/L, ≥ 30 min).

### Egg Viability

Eggs were collected and placed in a petri dish containing E3 embryo medium. Following an initial cleaning, brightfield images of plates containing eggs were taken using a Zeiss AxioZoom V.16 microscope and at the indicated timepoints thereafter. After imaging, nonviable eggs/embryos were immediately removed. Eggs/embryos were manually and blindly scored for viability using FIJI^12^.

### Microbiome Sample Processing and Sequencing

Following euthanasia, stool samples from resected guts were aseptically collected in ice-cold Dubelco’s PBS and pooled using a 5×3 pooling strategy (5 samples/replicate; n = 3 replicates/group with a total of n = 15 samples per group). Samples were spun and stored at −80°C prior to DNA isolation and 16s rRNA amplicon sequencing. DNA isolation, library preparation and sequencing were performed by the UNC Microbiome Core Facility. **DNA Isolation:** Samples were transferred to a 2 mL tube containing 200mg of 106/500μm glass beads (Sigma, St. Louis, MO) and 1 ml of Qiagen InhibitEX buffer (Hilden, Germany). The suspension was agitated for 10 minutes on a digital vortex mixer at 3000 rpm and incubated at 95°C for 5min, followed by agitation for 15 s on a Digital Vortex Mixer. After a 1 min centrifugation, the supernatant was transferred to a new tube containing 22.5 ul of Qiagen proteinase K, and 0.3 ml of Qiagen AL buffer was added, agitated on a digital vortex mixer for 15 s, and incubated at 70°C for 10 min. After a brief centrifugation, 0.3 ml of ethanol was added to the lysate and vortexed. DNA was purified using a standard on-column purification method with Qiagen buffers AW1 and AW2 as washing agents and eluted in DNase-free water^13–15^. **16S rRNA amplicon sequencing:** 12.5 ng of total DNA were amplified using universal primers targeting the V4 region of the bacterial 16S rRNA gene. Primer sequences contained overhang adapters appended to the 5’ end of each primer for compatibility with the Illumina sequencing platform. The primers used were F515/R806. Master mixes contained 12.5 ng of total DNA, 0.5 μM of each primer, and 2x KAPA HiFi HotStart ReadyMix (KAPA Biosystems, Wilmington, MA). The thermal profile for the amplification of each sample had an initial denaturing step at 95°C for 3 min, followed by cycling of denaturing at 95°C for 30 sec, annealing at 55°C for 30 sec, and a 30-second extension at 72°C (25 cycles), a 5-min extension at 72°C and a 4°C final hold. Each 16S amplicon was purified using the AMPure XP reagent (Beckman Coulter, Indianapolis, IN). In the next step, each sample was amplified using a limited cycle PCR program, adding Illumina sequencing adapters and dual-index barcodes (index 1(i7) and index 2(i5)) (Illumina, San Diego, CA) to the amplicon target. The thermal profile for the amplification of each sample had an initial denaturing step at 95°C for 3 min, followed by a denaturing cycle of 95°C for 30 sec, annealing at 55°C for 30 sec and a 30-sec extension at f72°C (8 cycles), a 5-min extension at 72°C and a final hold at 4°C. The final libraries were again purified using the AMPure XP reagent (Beckman Coulter), quantified, and normalized before pooling. The DNA library pool was then denatured with NaOH, diluted with hybridization buffer, and heat-denatured before loading on the NovaSeq reagent cartridge (Illumina) and the NovaSeq instrument (Illumina). Automated cluster generation and paired–end sequencing with dual reads were performed according to the manufacturer’s instructions^16^.

### Microbiome Bioinformatics Analysis

Sequencing output from the Illumina NextSeq2000 P2 PE300 were converted to fastq format and demultiplexed using Illumina BCL Convert 3.8.2–12 (Illumina, Inc.). The resulting paired-end reads were processed with the QIIME 2 2022 − 2^17,18^ wrapper for DADA2^19^ including merging paired ends, quality filtering, error correction, and chimera detection. Amplicon sequencing units from DADA2 were assigned taxonomic identifiers with respect to the Silva 138^20^ database. Alpha diversity with respect to Evenness index and Faith PD were estimated using QIIME 2 at a rarefaction depth of 5,000 sequences per subsample. Beta diversity estimates were calculated within QIIME 2 with respect to Weighted UniFrac and Bray Curtis dissimilarity between samples at a subsampling depth of 5,000. Results were summarized and visualized through principal coordinate analysis as implemented in QIIME 2. Aggregate differential abundance was estimated with ANCOM within QIIME 2 on genera with a minimum abundance of 5000 reads and minimum prevalence of 20%.

### RNA-seq

Following euthanasia, resected guts from n = 50 ~ 30dpf WT AB zebrafish were opened longitudinally, cleaned, minced with ultrafine surgical scissors prior to overnight storage in Buffer RLT (Qiagen) at −80°C. RNA was extracted using a Qiagen RNEasy kit according to manufacturer instructions. Samples were pooled using a 5×5 design such that each sample contained n = 5 extracts pooled equimolar with n = 5 samples per ELS and n = 5 samples per control group resulting in 600–1500ng total high-quality (A260/280 ~ 2.0) RNA extracts representing n = 25 extracts/group. QC/QC, mRNA library preparation and sequencing was performed by the UNC High Throughput Sequencing Core (HTSF). Briefly, mRNA library preparation was performed according to manufacturer-recommended protocols for mRNA using the Illumina TruSeq RNA Library Pep Kit v2 (Illumina, Inc., San Diego, California). Resulting libraries were sequenced on an Illumina NextSeq 2000 P2 flow cell using a paired end read format. RNA-seq Analysis was performed by ROSALIND, Inc. Pathway Analysis on the top 30 significantly differentially regulated genes was performed using the web-based GeneMANIA tool^21^ (Version 3.6.0).

### Statistics

GraphPad Prism (Version 10.5.0, GraphPad Software, LLC) was used for statistical comparisons and graphical representation. All data were tested for normality prior to statistical testing. T-test or ANOVA were used for statistical comparisons of two or more groups, respectively. Statistical analysis details can be found in each corresponding figure legend.

## Results

### ELS Reduces Egg Viability and Survivability

**Figure 2:**
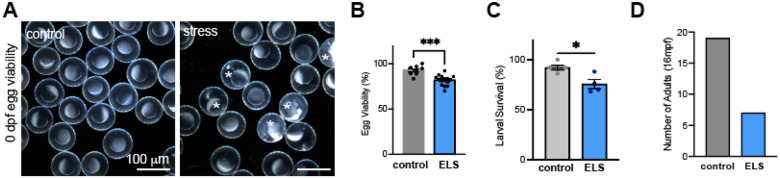
Reduced egg viability and survivability following ELS. (**A**) Brightfield microscope images of eggs from control (**A, left**) vs ELS-exposed breeders (**A, right**) * indicates nonviable eggs. (**B**) Quantification of egg viability expressed as percent viable of total eggs collected. (**C**) Survival of larval zebrafish reared from control compared to ELS-exposed fish. (**D**) Survival of adult offspring at 16 months post fertilization (mpf) comparing F3 generation control and ELS-exposed fish. *p ≤0.05; **p ≤ 0.01; ***p≤0.001 by Welch’s T-test.

ELS has been increasingly recognized as a factor that can negatively impact egg quality and reproductive viability in animal models and human studies^22–26^. In line with this, we noticed qualitative differences in egg viability between breeders previously exposed to chronic ELS compared to unexposed sibling controls (Fig. 2A). Upon quantification, we found that fish exposed to ELS during larval development produced significantly fewer viable eggs in adulthood (Fig. 2B). Further, even after standardization of viable embryos/larvae, we found that fewer fish survive to larval transition (Fig. 2C). In addition to reduced viability of eggs and reduced survival of embryos and larvae, we also observed that survival to adulthood was significantly impaired in fish exposed to ELS (63% survivability in controls vs. 23% survivability in ELS) suggesting that ELS induces long-lasting physiological changes affecting both reproduction and viability that persist throughout life and into adulthood (Fig. 2D).

### Effects of ELS on Gut Microbiome

**Figure 3:**
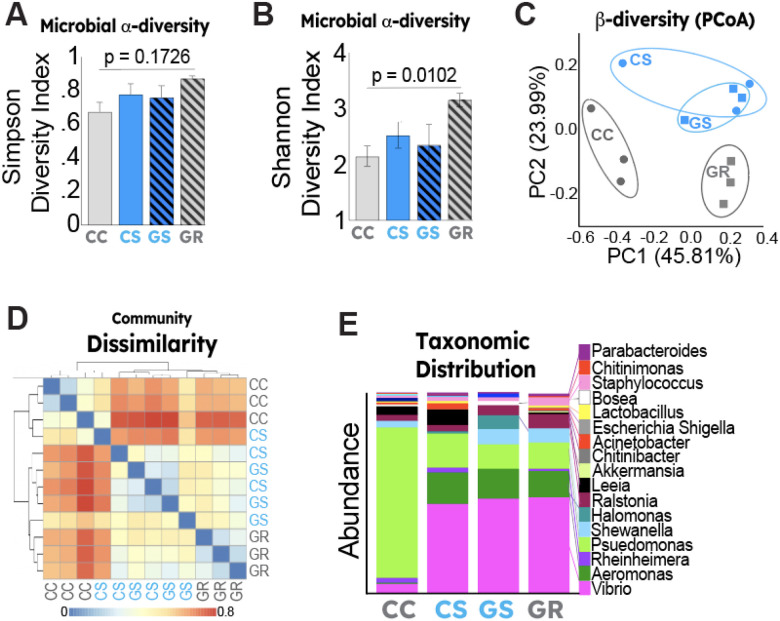
Gut microbiota effects following generational early life stress. Microbial a-diversity shown as (**A**) Simpson or (**B**) Shannon Diversity Indices. (**C**) b-diversity shown as a two-dimensional principal component analysis (PCoA). Each dot represents one pooled sample (n=5/sample) (**D**) Community dissimilarity profiles shown as Bray-Curtis Index between control (CC), ELS (CS), generational stress (GS) or generational recovery (GR) fish. (**E**) Shows taxonomic distribution of microbial communities at the genus level.

Recent studies have demonstrated a link between gut microbial composition, reproductive function, and fertility^9^. In addition, ELS has been shown to impact the gut microbiota in diverse species^2–4,27,28^. To determine whether ELS impacted gut microbial architecture in developing zebrafish, we conducted 16S rRNA microbial sequencing on stool samples of zebrafish exposed to ELS both within-generation and from an intergenerational context. We first interrogated microbiome alpha-diversity and found no significant differences between control vs. ELS fish (Fig. 3A). Interestingly, zebrafish with generational exposure to ELS but without intragenerational exposure (“Generational Recovery” group), we found a significant increase in the Shannon Diversity Index (Fig. 3B). Principal Component and Bray-Curtis Analysis revealed a distinct separation of the control group without generational stress exposure (CC) and the generational recovery group (GR), with significant overlap between the ELS-exposed groups (both with and without generational stress exposure) (Fig. 3C–D). An analysis of taxonomic distribution at the genus level revealed distinct separation of the control group (CC) compared to that of ELS-exposed fish from either the within-generation or intergenerational context (Fig. 3E). Specifically, we observed that control animals had significantly increased presence of *Pseudomonas spp*. compared to ELS-exposed fish which had an expansion of *Vibrio* and *Aeromonas* spp. In the generationally exposed groups (GS and GR), we also observed an increase in *Shewanella* spp.

### Effects of ELS on Gut Transcriptome

**Figure 4:**
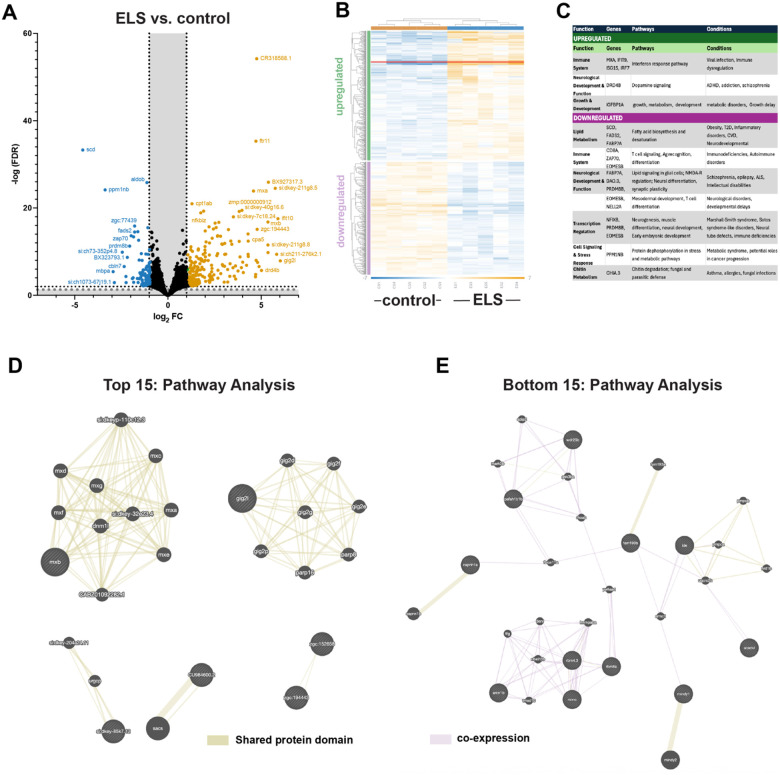
ELS alters the transcriptional profile of the zebrafish gut. Differentially expressed genes in ELS vs. control zebrafish gut shown as a (**A**) Volcano plot or (**B**) Heatmap. The colors in the heatmap are defined as a blue-white-orange gradient. Each rectangle represents mean data from n=5 fish. (**C**) Table of functional analysis of top pathways. GeneMANIA Pathway Analysis showing top 15 upregulated (**D**) and top 15 downregulated (**E**) genes.

Our previous study revealed that ELS altered the neuroimmune profile and functioning of the developing zebrafish gut^1^. In this study, we adopted an unbiased approach to determine whether ELS and/or ELS-associated microbial dysbiosis altered the transcriptional landscape of the developing gut (Fig. 4A). We found that ELS was associated with the significant differential expression of 830 genes (Fig. 4B). Although many of the genes were undefined (Fig. 4A), we found that many upregulated genes were associated with immune system function and interferon response pathways and many downregulated genes are involved in lipid metabolism, T cell signaling, neural differentiation and transcriptional regulation (Fig. 4C). We performed a pathway analysis and found significant engagement of myxovirus resistance (mxb) and *gig2* genes, which was first identified as a fish interferon-stimulated gene (Fig. 4D). Taken together these data suggest that ELS-exposed fish upregulate gut anti-microbial response pathways, either as a direct result of ELS or due to increased susceptibility to infection and microbial dysbiosis.

## Discussion

Early life stress is increasingly associated with pathological changes to the brain-gut-microbiota axis^29–32^. Additionally, ELS has been associated with changes to the gonadal axis, affecting fertility, reproduction, growth, and survival^33,34^. Despite these compelling associations, the underlying mechanisms mediating pathological consequences to major bodily systems later in life remain to be elucidated. In this study, we report observations that ELS reduces egg viability and survival and results in major changes to the gut microbiota through a generational lens. Additionally, we interrogated potential host mechanisms using an unbiased, whole-tissue transcriptional approach.

In line with previous studies, we found that chronic ELS experienced prior to juvenile-to-adult transition reduces the egg quality of zebrafish later in adulthood. We also observed reduced survival of larval offspring following ELS which persists throughout adulthood resulting in significantly reduced survival to adulthood (Fig. 2).

Stress has been found to alter the composition of the gut microbiota in humans and model systems including rodents and fish^22–26^. In this study, we report that ELS induces profound and persistent shifts in the microbial composition of the developing zebrafish gut. Although intragenerational ELS exposure was not associated with significant changes to microbial a-diversity, we found that the “generational recovery” group exhibited a significantly increased bacterial a-diversity as compared with intra-generationally exposed (CS or GS) or the control (CC) groups (Fig. 3). Although increased gut microbial diversity has been broadly associated with organismal health^35^, recent studies argue that gut microbial a-diversity does not reliably indicate organismal healthiness.^36^ We also observed changes to b-diversity and community composition with strong dissimilarly profiles with no overlap between the control (CC) and recovery (GR) groups while stress groups (CS and GS) exhibited significant overlap. Taken together these data indicate that ELS results in unique microbial signatures that partially persists to subsequent generations.

Intriguingly, control zebrafish without prior generational ELS exposure exhibited a striking dominance of *Pseudomonas* spp. In contrast, in zebrafish exposed to within- or intergenerational ELS (CS, GS, and GR), we observed a significant expansion in *Aeromonas* and *Vibrio* spp (Fig. 3E). *Aeromonas* colonization has been shown to disrupt microbial composition, reducing beneficial bacteria and promoting growth of pathogenic spp. resulting in their categorization as opportunistic pathogens^37^. Similarly, *Vibrio* spp., although ubiquitous in aquatic environments, has been shown to opportunistically infect zebrafish^38^. In zebrafish generationally exposed to ELS with or without within-generation ELS exposure, we also observed an increase in *Shewanella* spp. that was not observed in the control or within-generation ELS exposure groups (CC and CS). Although *Shewanella* spp. are a considered to be a constituent of the core intestinal microbiota of zebrafish, they can also be considered opportunistic pathogens. These data suggest that ELS confers infection susceptibility in developing zebrafish. Whole-gut transcriptional profiling revealed that ELS exposure results in dramatic changes to transcriptional signaling in the gut which was associated with increased transcription of interferon-stimulated genes (Fig. 4).

Altogether our data suggest that ELS exposure renders developing zebrafish susceptible to opportunistic infection by potentially pathogenic species and that offspring of stressed animals continue to remain susceptible to opportunistic infection, potentially affecting viability and fitness of offspring through modulation of host-microbial interactions.

## Data Availability

All data referenced in this manuscript will be made immediately available upon reasonable request. Data requests should be made directly to the communicating author. The datasets generated during this study are available through the NIH National Library of Medicine, BioProject ID: PRJNA1337436.

## Highlights

- Early life stress reduces egg viability, larval, and adult survival
- Early life stress alters gut microbial composition and diversity
- Early life stress induces profound transcriptional changes in the developing gut
